# Structural Roles for the Juxtamembrane Linker Region and Transmembrane Region of Synaptobrevin 2 in Membrane Fusion

**DOI:** 10.3389/fcell.2020.609708

**Published:** 2021-01-06

**Authors:** Yaru Hu, Le Zhu, Cong Ma

**Affiliations:** Key Laboratory of Molecular Biophysics of the Ministry of Education, College of Life Science and Technology, Huazhong University of Science and Technology, Wuhan, China

**Keywords:** synaptobrevin-2, SNARE complex assembly, membrane fusion, Munc18, Munc13

## Abstract

Formation of the *trans*-SNARE complex is believed to generate a force transfer to the membranes to promote membrane fusion, but the underlying mechanism remains elusive. In this study, we show that helix-breaking and/or length-increasing insertions in the juxtamembrane linker region of synaptobrevin-2 exert diverse effects on liposome fusion, in a manner dependent on the insertion position relative to the two conserved tryptophan residues (W^89^/W^90^). Helical extension of synaptobrevin-2 to W^89^/W^90^ is a prerequisite for initiating membrane merger. The transmembrane region of synaptobrevin-2 enables proper localization of W^89^/W^90^ at the membrane interface to gate force transfer. Besides, our data indicate that the SNARE regulatory components Munc18-1 and Munc13-1 impose liposome fusion strong demand on tight coupling between the SNARE motif and the transmembrane region of synaptobrevin-2.

## Introduction

Neurotransmitter release mediated by synaptic exocytosis requires the fusion of synaptic vesicles with the plasma membrane of nerve cells. To accomplish fusion, membranes must overcome the energy barriers created by charge repulsing, local dehydration of polar phospholipid headgroups and membrane deformation. In synaptic exocytosis, the synaptic vesicle SNARE protein synaptobrevin-2 (R-SNARE) assembles with the plasma membrane SNARE proteins SNAP-25 and syntaxin-1 (both referred to as Q-SNAREs) to form the SNARE complex to catalyze the fusion of the two membranes (Südhof and Rizo, 2011; Jahn and Fasshauer, 2012; Han et al., 2017). In addition, a number of accessory proteins are required for the exquisite regulation of SNARE complex formation and SNARE-mediated membrane fusion (Rizo and Xu, 2015; Brunger et al., 2019).

SNARE complex assembly is characterized by the formation of a parallel four-helix bundle composed of four SNARE motifs, with synaptobrevin-2 and syntaxin-1 each contributing one motif whereas SNAP-25 contributes two (Fasshauer et al., 1998; Sutton et al., 1998). In synaptobrevin-2 and syntaxin-1, the SNARE motif is connected by juxtamembrane linker region (JLR) to C-terminal transmembrane region (TMR) that anchor at the vesicles and the plasma membrane, respectively. SNAP-25 is anchored to the plasma membrane by palmitoyl chains bound to cysteine residues in a loop region connecting its two SNARE motifs. The assembly of a “*trans*” four-helix bundle upon an N- to C-zippering mode releases energy to bring the membranes into close proximity (Chen and Scheller, 2001; Jahn et al., 2003; Sørensen et al., 2006). Subsequent assembly proceeding over the JLR and TMR of synaptobrevin-2 and syntaxin-1 is believed to transmit the energy into the membrane interface, leading to a conversion from the “*trans*” complex into a “*cis*” complex in which the two TMRs are aligned in parallel in the same membrane (Han et al., 2017). This configuration transition is assumed to be functional at the final stage of exocytosis by facilitating membrane deformation and expansion of the fusion pore.

A variety of SNARE-based reconstitution experiments *in vitro* have demonstrated that efficient membrane fusion requires the SNARE motifs to support “*trans*” complex assembly, and the JLRs and TMRs to drive “*cis*” complex formation (Han et al., 2017). A solved crystal structure of the neuronal SNARE complex showed that assembly proceeds over the SNARE four-helix bundle, resulting in a continuous helical bundle extending to the end of the JLRs and TMRs (Stein et al., 2009). This study suggests a helical continuity model in which membrane fusion requires assembly of the SNARE complex all the way into the membranes. Although this model appears to be structurally and energetically attractive, a number of studies have challenged the model. For instance, helix-breaking mutations in the JLRs had little influence on fusion *in vitro* (Mcnew et al., 1999; Van Komen et al., 2005; Pieren et al., 2015) Similarly, a synaptobrevin-2 mutant carrying two helix-disrupting proline residues in the JLR enabled a nearly complete rescue of fusion in chromaffin cells (Kesavan et al., 2007). These results challenged the notion that the helical continuity model underlies the common nature of the general fusion mechanism. On the other hand, increasing length and flexibility of the JLRs by amino acid insertions gradually decrease fusion efficiency but does not eliminate fusion *in vitro* (Mcnew et al., 1999). Strikingly, synaptobrevin-2 with a 12-residue insertion or syntaxin-1 with a 7-residue insertion in the JLR was found to completely restore spontaneous release in cultured neurons (Deak et al., 2006; Zhou et al., 2013). Despite such mild effect on spontaneous fusion, synaptobrevin-2 or syntaxin-1 with a 3-residue (or 4-residue) insertion in the JLR were observed to dramatically reduce the RRP size and Ca^2+^-evoked exocytosis in cultured neuronal and chromaffin cells (Kesavan et al., 2007; Guzman et al., 2010; Borisovska et al., 2012; Zhou et al., 2013). These data suggest that evoked fusion but not spontaneous fusion requires an extremely tight coupling between the SNARE motifs and the TMRs.

Although early studies have established the essential role of the TMRs in membrane fusion (Giraudo et al., 2005; Hofmann et al., 2006; Chang et al., 2016; Dhara et al., 2016; Chiang et al., 2018), this issue has become controversial. Previous work on yeast vacuolar fusion found that lipid-anchored R-SNARE Nyv1p, which lacks the TMR, supports fusion in the presence of the HOPS tethering complex that includes the Sec1/Munc18 (SM) protein Vps33p (Xu et al., 2011). Similarly, a more recent study on lytic granule exocytosis indicated that the SM protein Munc18-2 can assist lipid-anchored syntaxin-11 (without the TMR) to drive complete membrane fusion (Spessott et al., 2017). Consistent with these observations, it was found that lipid-anchored SNAREs (sytnaxin-1 and synaptobrevin-2) without the TMRs can totally rescue spontaneous and partially rescue Ca^2+^-evoked release in neurons (Zhou et al., 2013). The dispensable role of the TMR in these studies challenged the helical continuity model, raising a possibility that SNARE complex assembly may be sufficient to destabilize the phospholipid membrane and induce full fusion, in a manner upon forcing the two opposing membrane into close proximity but without a need for the TMR. In this scenario, the contribution of the SNARE regulatory components (e.g., SM proteins and tethering factors that regulate SNARE complex assembly) in facilitating this process has been unclear and need to be investigated.

Indeed, in synaptic exocytosis, SNARE-mediated membrane fusion is highly regulated by the SM protein Munc18-1 and the tethering-related protein Munc13s (Rizo and Xu, 2015; Brunger et al., 2019). A wealth of evidence revealed that Munc18-1 and Munc13s cooperate to promote fusion via chaperoning proper SNARE assembly (Ma et al., 2013; Lai et al., 2017; Wang et al., 2019) and via protecting the assembled SNARE complex against NSF/α-SNAP disassembly (Ma et al., 2013; He et al., 2017; Jakhanwal et al., 2017). Besides, Munc18-1 and Munc13s have been reported to associate with the membranes via their intrinsic membrane-binding sites independent of the existence of the SNAREs (Xu et al., 2011; Quade et al., 2019). Despite these findings, it remains unclear whether Munc18-1 and Munc13s could act as force generators that exert direct force to induce membrane deformation and fusion, in addition to their regulatory role in SNARE complex assembly.

Here, we have systematically investigated the roles of the synaptobrevin-2 JLR and TMR in membrane fusion using *in-vitro* reconstitution systems with and without Munc18-1 and Munc13-1. Our data showed that helical extension of synaptobrevin-2 to the two consecutive tryptophan residues (W^89^/W^90^) in the JLR is absolutely required for initiating membrane merger, and helical extension beyond W^89^/W^90^ is crucial for complete membrane fusion. Membrane-embedded TMR directs W^89^/W^90^ to position at the membrane-water interface, enabling W^89^/W^90^ to gate force transfer as a fusion barrier. Besides, our data indicate that Munc18-1 and Munc13-1 impose liposome fusion strong demand on tight coupling between the SNARE motifs and the TMR.

## Results

### Fusion Affected by Disrupting Helical Continuity of Synaptobrevin-2

**Figure 1:**
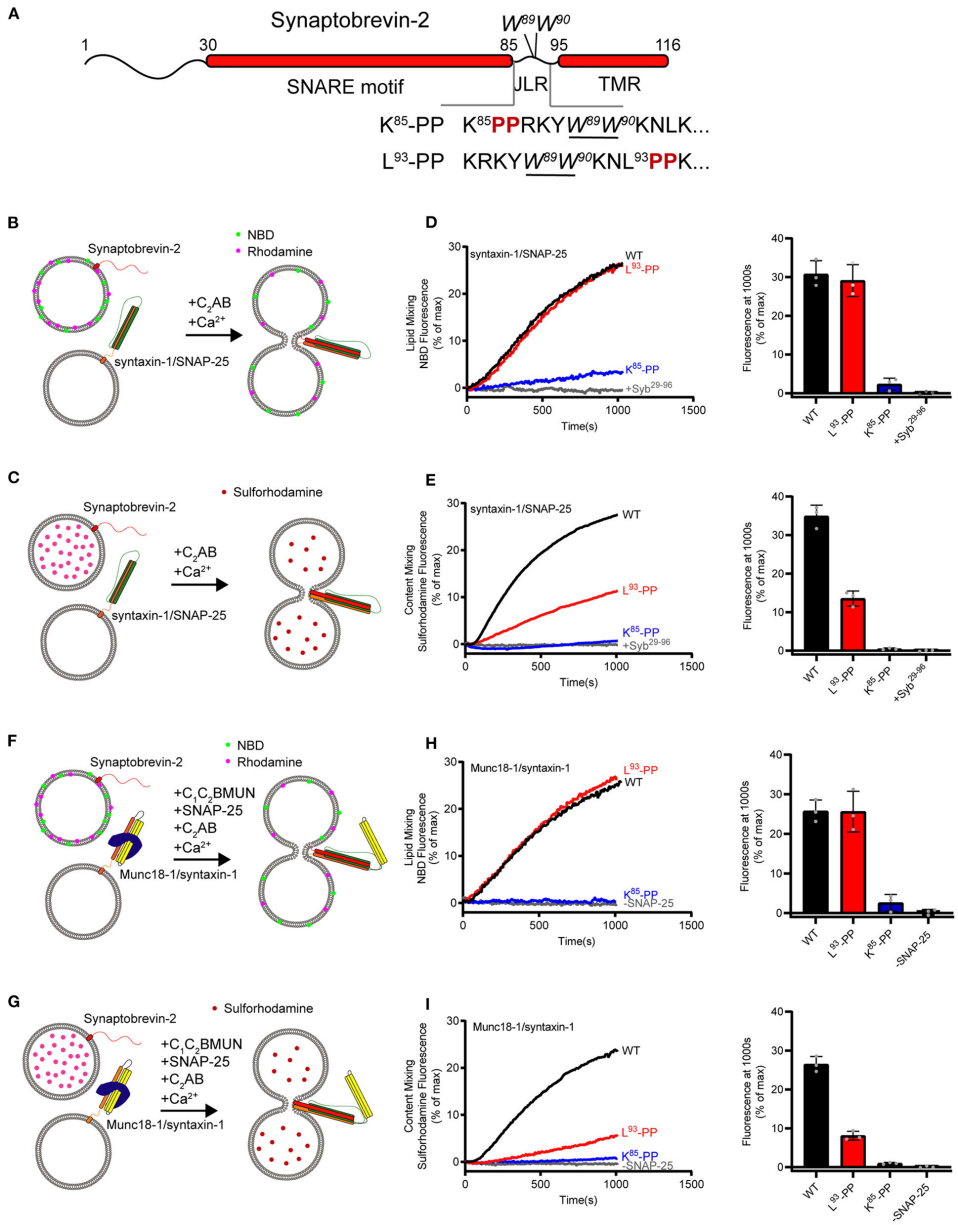
Fusion affected by disrupting helical continuity of the synaptobrevin-2. **(A)** Domain structure of full length wild-type synaptobrevin-2 (WT) and its mutants with two-proline insertions after K^85^ (K^85^-PP) or L^93^ (L^93^-PP) in the JLR. **(B,C)** Scheme of lipid mixing **(B)** and content mixing assay **(C)** between syntaxin-1/SNAP-25 and synaptobrevin-2 liposomes in the presence of C_2_AB fragment and 1 mM Ca^2+^. Note that the syntaxin-1 SNARE motif (H3, residues 183–288) was used here to form the syntaxin-1/SNAP-25 complex. **(D,E)** Lipid **(D)** and content mixing **(E)** of synaptobrevin-2 WT, K^85^-PP, and L^93^-PP liposomes with syntaxin-1/SNAP-25 liposomes. **(F,G)** Scheme of lipid mixing **(F)** and content mixing assay **(G)** between Munc18-1/syntaxin-1 (full length, residues 1–288) and synaptobrevin-2 liposomes in the presence of the Munc13-1 C_1_-C_2_B-MUN fragment, SNAP-25, C_2_AB fragment, and 1 mM Ca^2+^. **(H,I)** Lipid **(H)** and content mixing **(I)** of synaptobrevin-2 WT, K^85^-PP, and L^93^-PP liposomes with Munc18-1/syntaxin-1 liposomes. Representative traces came from one of three independent experiments. Bars on the right panel in **(D,E,H,I)** are means ± SDs, *n* = 3.

The JLR of synaptobrevin-2 contains two consecutive tryptophan residues (W^89^/W^90^), which are proposed to localize at the membrane-water interface and serve as a fusion barrier (Chen et al., 2004; Borisovska et al., 2012). Earlier studies showed that mutation or insertion of two helix-breaking proline residues in the JLR of synaptobrevin-2 has little effect on membrane fusion and exocytosis (Mcnew et al., 1999; Van Komen et al., 2005), at odds with the helical continuity model (Stein et al., 2009). However, it is noteworthy that these mutations or insertions were placed downstream of W^89^/W^90^, leading us to doubt whether insertion of helix-breaking residues upstream of W^89^/W^90^ would exert more strong effect. Hence, we placed two proline residues symmetrically on either side of W^89^/W^90^, i.e., (K^85^-PP) and (L^93^-PP), respectively (Figure 1A), and examined their effects on membrane fusion.

First, we assayed fusion between liposomes harboring the syntaxin-1/SNAP-25 complex and liposomes bearing synaptobrevin-2 (wild type, WT) or its two mutations (K^85^-PP and L^93^-PP). Co-flotation assay confirmed that synaptobrevin-2 and its mutants were reconstituted on liposomes (Supplementary Figures 1A,B). To explore membrane fusion, we performed lipid-mixing and content-mixing assays (Figures 1B,C), as previously described (Weber et al., 1998; Wang et al., 2016). Note that the cytoplasmic fragment of synaptotagmin-1 (C_2_AB) and Ca^2+^ were included to enhance fusion rate and extent throughout this study. As expected, WT synaptobrevin-2 supported efficient lipid mixing and content mixing (Figures 1D,E). As a control, the addition of the cytoplasmic fragment of synaptobrevin-2 (Syb^29−96^) abolished both lipid mixing and content mixing (Figures 1D,E). L^93^-PP designed to disrupt helical continuity downstream of W^89^/W^90^ supported lipid mixing as effectively as WT synaptobrevin-2, but reduced content mixing by 55% compared to WT synaptobrevin-2 (Figures 1D,E). Intriguingly, K^85^-PP proposed to break helical continuity upstream of W^89^/W^90^ severely impaired both lipid and content mixing (Figures 1D,E). These data led to an idea that helical extension of synaptobrevin-2 to W^89^/W^90^ in the JLR is essential for initiating membrane merger, and helical continuity beyond W^89^/W^90^ contributes to drive complete membrane fusion.

To investigate whether the SNARE regulatory components Munc18-1 and Munc13-1 influence the dependence of SNARE-mediated liposome fusion on helical continuity of synaptobrevin-2, we exploited Munc18–Munc13-regulated fusion system wherein Munc13-1 catalyzes SNARE complex formation starting from the Munc18-1/syntaxin-1 complex (Ma et al., 2013). Hence, we assayed both lipid mixing and content mixing between liposomes bearing the Munc18-1/syntaxin-1 complex and liposomes containing synaptobrevin-2 and its mutations (K^85^-PP and L^93^-PP) in the presence of SNAP-25, C_1_-C_2_B-MUN (containing the priming activity of Munc13-1), C_2_AB and Ca^2+^ (Figures 1F,G), as previous described (Ma et al., 2013; Yang et al., 2015). As a control, lipid mixing and content mixing were abolished when SNAP-25 was absent (Figures 1H,I). Intriguingly, K^85^-PP abrogated both lipid mixing and content mixing, while L^93^-PP effectively supported lipid mixing but reduced content mixing by 70% (Figures 1H,I), in line with the results obtained with the reconstitution system deficient in Munc18-1 and Munc13-1 (Figures 1B–E).

In addition to K^85^-PP and L^93^-PP, we introduced another two proline-insertion mutations that are more adjacent to W^89^/W^90^ of synaptobrevin-2, referred to as Y^88^-PP and W^90^-PP, respectively (Supplementary Figure 2A). Co-flotation experiment confirmed that Y^88^-PP and W^90^-PP were reconstituted on liposomes as effective as WT synaptobrevin-2 (Supplementary Figure 1B). Consistently, using systems with and without Munc18-1 and Munc13-1, we observed that Y^88^-PP and W^90^-PP exhibited distinct effect on lipid and content mixing, in a manner similar to K^85^-PP and L^93^-PP, respectively (Supplementary Figures 2B–E). Notably, we verified that the content mixing assay in both systems reflects actual exchange of content between liposomes but not the leakage of sulforhodamine (Supplementary Figures 2F,G).

Taken together, the data obtained from both fusion systems with and without Munc18-1 and Munc13-1 suggest that helical continuity of synaptobrevin-2 extended to W^89^/W^90^ in the JLR is an essential prerequisite to initiate membrane merger. The data indicate that W^89^/W^90^ functions as a fusion landmark that gates force transmission into the membrane interface.

### Fusion Affected by Substitution or Deletion of the Synaptobrevin-2 TMR

**Figure 2:**
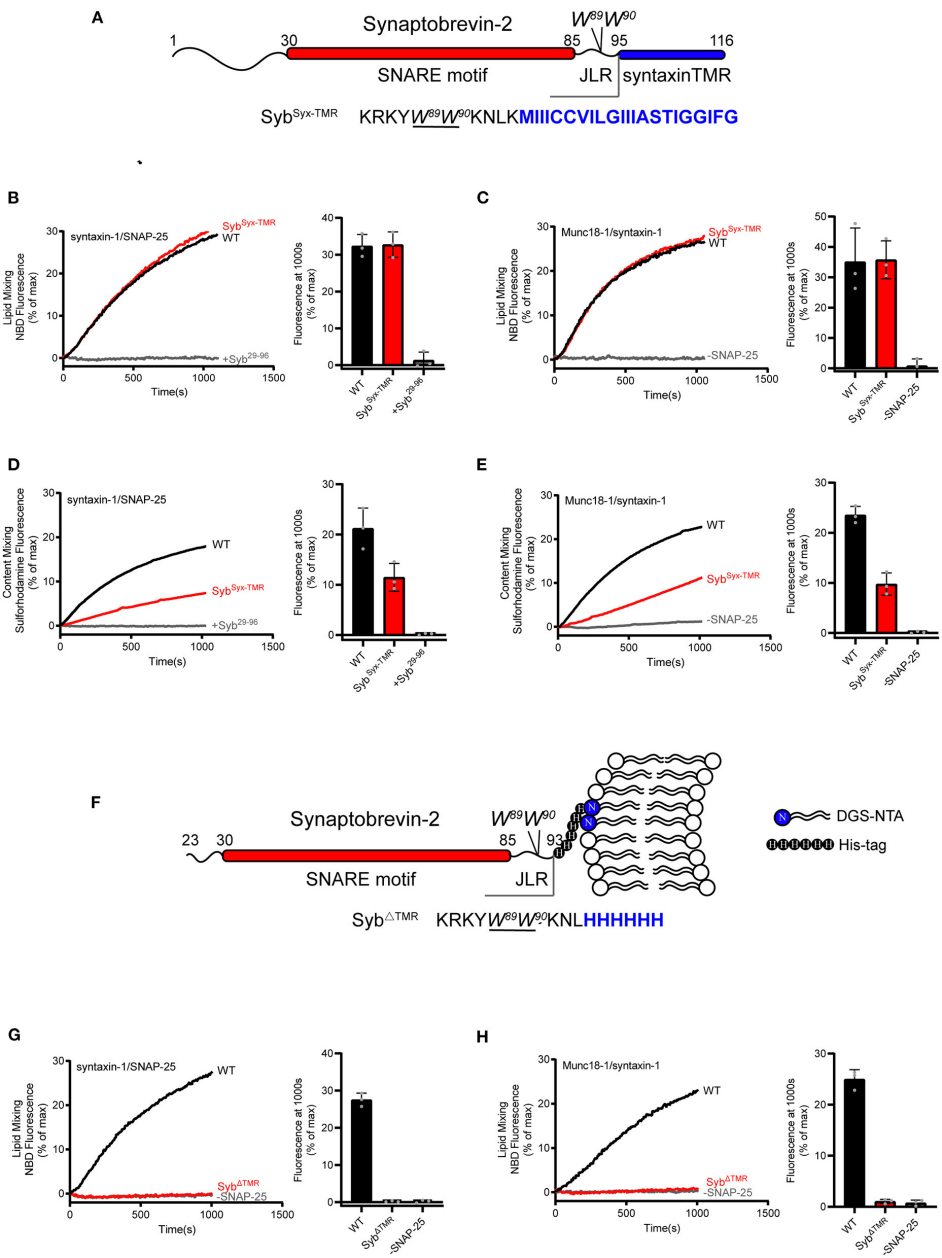
Fusion affected by substitution or deletion of the synaptobrevin-2 TMR. **(A)** Domain structure of chimeric synaptobrevin-2 with TMR substituted by syntaxin-1 TMR (residues 267–288) (Syb^Syx−TMR^). **(B,C)** Lipid mixing between synaptobrevin-2 WT, Syb^Syx−TMR^ liposomes and liposomes reconstituted with syntaxin-1/SNAP-25 **(B)** or Munc18-1/syntaxin-1 **(C)**. **(D,E)** Content mixing between synaptobrevin-2 WT, Syb^Syx−TMR^ liposomes, and liposomes reconstituted with syntaxin-1/SNAP-25 (**D**) or Munc18-1/syntaxin-1 **(E)**. **(F)** Domain structure of TMR deletion mutant (Syb^ΔTMR^) with C-teminal 6-Histidine tag linked to DGS-NTA-containing liposome. **(G,H)** Lipid mixing between synaptobrevin-2 WT and Syb^ΔTMR^ liposomes and liposomes reconstituted with syntaxin-1/SNAP-25 **(G)** and Munc18-1/syntaxin-1 **(H)**. Representative traces came from one of three independent experiments. Bars on the right panel in **(B–E,G,H)** are Means ± SDs, *n* = 3.

Next, we investigated for the importance of the synaptobrevin-2 TMR in membrane fusion with both fusion systems described above. In this respect, we generated a synaptobrevin-2 mutant that lacks its own TMR but bears the syntaxin-1 TMR, referred to as Syb^Syx−TMR^, with the sequence following W^89^/W^90^ (residues 95–116) in synaptobrevin-2 replaced with the TMR sequence (residues 267–288) of syntaxin-1 (Figure 2A). Co-flotation experiment confirmed that Syb^Syx−TMR^ was reconstituted on liposomes as effectively as WT synaptobrevin-2 (Supplementary Figure 1C). In both fusion systems with and without Munc18-1 and Munc13-1, Syb^Syx−TMR^ supported lipid mixing (Figures 2B,C) but obviously impaired content mixing by 50% and 55%, respectively (Figures 2D,E), implying that content mixing requires more precise sequence and topology structure of the TMR.

As the role of the TMR of synaptobrevin-2 in lipid mixing can be fully compensated by the TMR of syntaxin-1 (Figures 2B,C), we doubted whether the TMR is really required for initiating membrane merger. To explore this, we generated a TMR-deleted synaptobrevin-2 mutant (residues 23–93) and made this mutant attach the membrane surface upon introducing a 6-Histidine tag to its C-terminal end (after the residue L^93^, referred to as Syb^ΔTMR^), and examined its activity in membrane fusion (Figure 2F). Five percentage molar ratio of DGS-NTA was accordingly included in liposomes (Figure 2F). Co-flotation assay verified efficient attachment of Syb^ΔTMR^ to liposomes (Supplementary Figure 1C). By using a Syb^49−96^ peptide displacement experiment (Pobbati et al., 2006), we found that Syb^ΔTMR^ was capable of forming *trans*-SNARE complexes with the syntaxin-1/SNAP-25 complex between the two membranes (Supplementary Figure 3). However, in comparison to WT synaptobrevin-2, Syb^ΔTMR^ failed to support lipid mixing (Figures 2G,H) in the absence and presence of Munc18-1 and Munc13-1. Hence, despite that Syb^ΔTMR^ can bring the membranes into close distance, the loss of lipid mixing by the lack of the TMR cannot be compensated by Munc18-1 and Munc13-1. These data, together with the results observed in Figure 1, indicate that even in a condition that the two membranes have reached to a close proximity, Munc18-1 and Munc13-1 execute no sufficient force or energy to induce membrane deformation and fusion, in addition to their important regulatory function in SNARE complex assembly.

**Figure 3:**
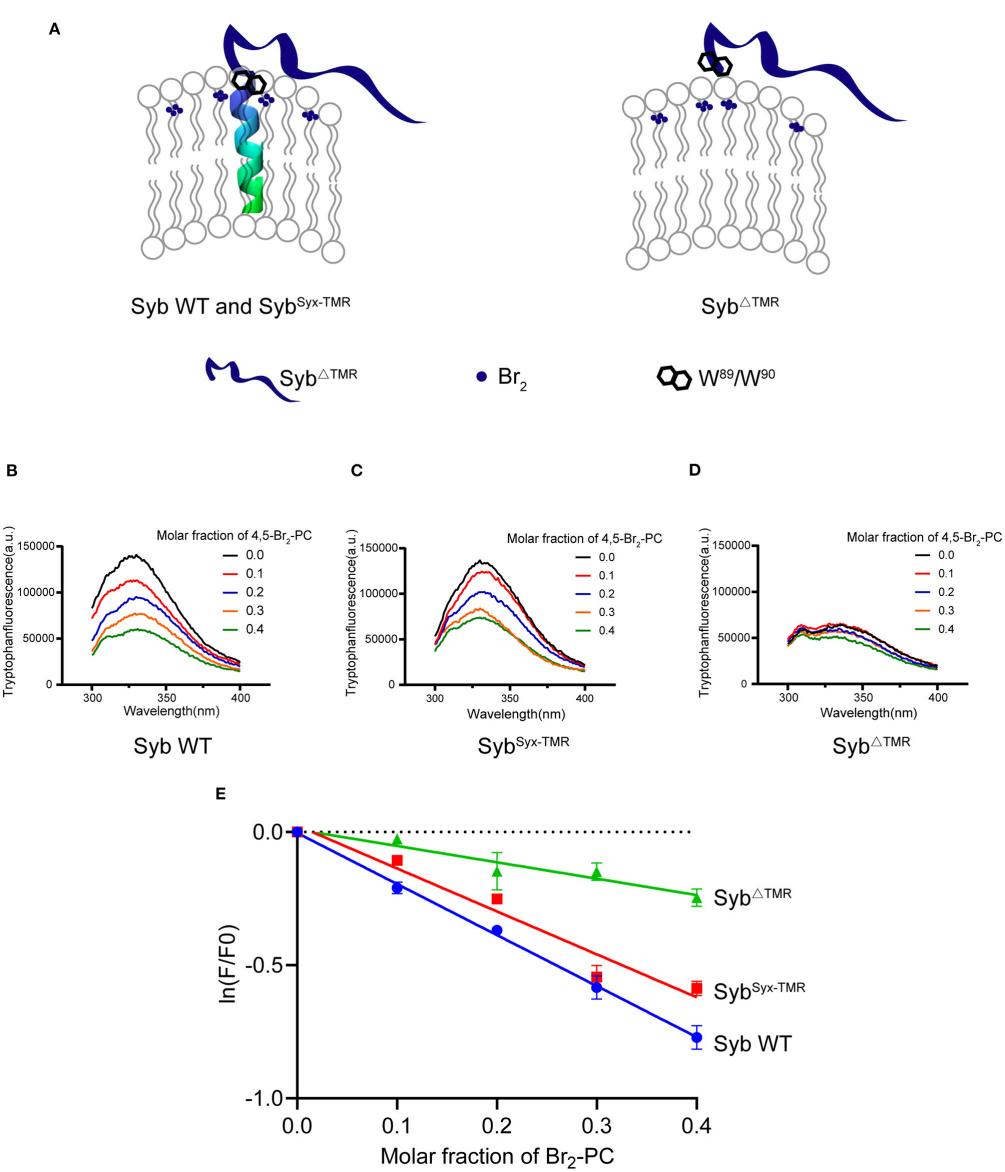
W^89^/W^90^ of TMR anchored synaptobrevin-2 are resided at the membrane-water interface. **(A)** Schematic diagrams of lipid quenching assay showing that fluorescence of embedded tryptophans in TMR anchored synaptobrevin-2 were quenched by lipid quencher 4,5-Br_2_-PC. **(B–D)** Quenching of tryptophan fluorescence by different molar fraction of 4,5-Br_2_-PC in synaptobrevin-2 WT **(B)**, Syb^Syx−TMR^ **(C)**, and Syb^ΔTMR^ **(D)** liposomes. The samples were excited at 285 nm, and the emission spectra were collected in the range of 300–400 nm. **(E)** Quantification of the fluorescent intensities in **(B–D)** of synaptobrevin-2 WT (solid blue circles), Syb^Syx−TMR^ (solid red squares), and Syb^ΔTMR^ (solid green triangles) liposome. The total fluorescence intensity (*F*) was calculated by integrating the intensity in the emission spectral range. (*F*_0_) represents the fluorescent intensity in the absence of 4,5-Br_2_-PC, ln (*F/F*_0_) is plotted against the 4,5-Br_2_-PC molar fraction. Linear regression was performed by Prism 6.01. Bars in **(E)** are presented as Means ± SDs, *n* = 3.

Since the synaptobrevin-2 mutants described above, e.g., L^93^-PP, W^90^-PP, Syb^Syx−TMR^, and Syb^ΔTMR^, are able to anchor on the liposomes and carry the W^89^/W^90^ residues, how to interpret that only Syb^ΔTMR^ fails to support lipid mixing? It was suggested that precise insertion of the W^89^/W^90^ residues at the membrane-water interface is essential for fusion (Borisovska et al., 2012). We thus examined the position of the W^89^/W^90^ region of the mutants with respect to the membrane-water interface. To this aim, we applied brominated lipid into synaptobrevin-2-liposomes where bromines were attached to the acyl chain (4,5-Br_2_-PC) (Figure 3A). In this case, tryptophan fluorescence (*F*) could be effectively quenched if the W^89^/W^90^ residues were in contact with the acyl chain (Kweon et al., 2003). Indeed, a significant decrease in *F* was observed for WT synaptobrevin-2 with increased mole fraction of Br_2_-PC (Figures 3B,E), confirming the close contact of the W^89^/W^90^ residues with the membrane. Similar result was observed for Syb^Syx−TMR^ (Figures 3C,E), indicating that substitution of the TMR of synaptobrevin-2 with that of syntaxin-1 retains the ability to bring the W^89^/W^90^ residues at the membrane surface. However, little decrease in *F* was detected for Syb^ΔTMR^ (Figures 3D,E), suggesting that the two tryptophan residues are sequestered from the membrane-water phase owing to the lack of the TMR. Control experiments ruled out the possibilities that 5% molar ratio of DGS-NTA quenches tryptophan fluorescence (Supplementary Figure 4A) and that Ca^2+^ competes with DGS-NTA for Syb^ΔTMR^ binding (Supplementary Figure 1C). Moreover, based on brominated lipid tryptophan quenching assay, we found that the behaviors of the tryptophans of Y^88^-PP, W^90^-PP, K^85^-9i, and L^93^-9i are similar to that of WT synaptobrevin-2 (Supplementary Figure 4B). These data showed that tryptophan fluorescence is insensitive to the PP and Gly/Ser insertions, implying that the insertions do not change position or orientation of W^89^/W^90^ with respect to the membrane.

Thus, we suggest that although Syb^ΔTMR^ retains the ability to attach on the membrane surface, improper position, or orientation of W^89^/W^90^ with respect to the membrane caused by deletion of the TMR is expected to impair the force-transmission role of W^89^/W^90^.

### Fusion Affected by Extension of the Length or Flexibility of the Synaptobrevin-2 JLR

Previous studies have found that extension of the length or flexibility of the synaptobrevin-2 JLR exerts milder effects on spontaneous release but severe impairment on Ca^2+^-evoked release (Deak et al., 2006; Bretou et al., 2008; Guzman et al., 2010; Zhou et al., 2013), suggesting that a tight coupling between the SNARE motif and the TMR is required for highly regulated fusion events. We hence explored whether such tight coupling demand is imposed by the SNARE regulatory proteins.

**Figure 4:**
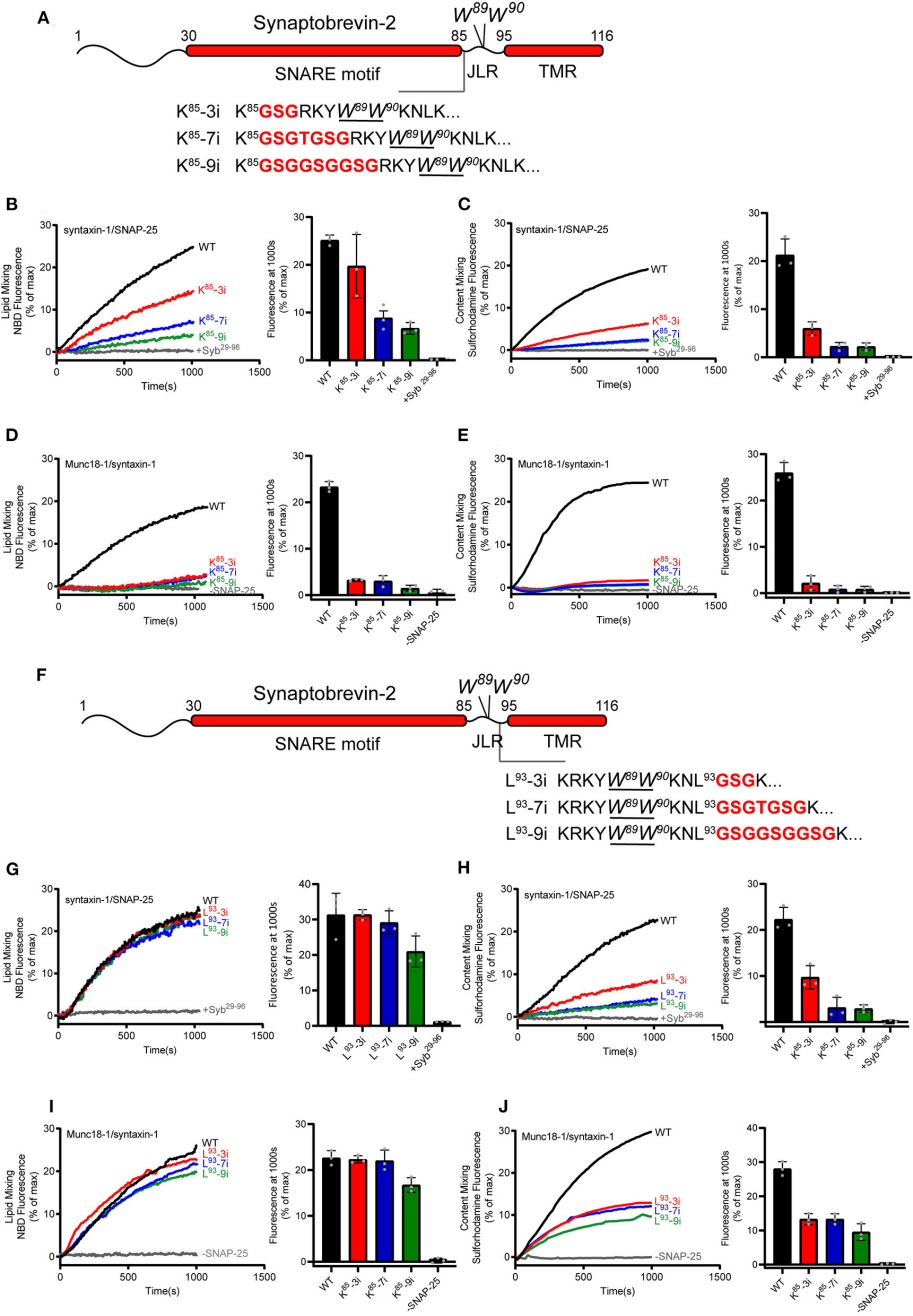
Fusion affected by extension of the length or flexibility of the synaptobrevin-2 JLR. **(A)** Domain structure of full length synaptobrevin-2 with 3, 7, 9 GSG insertions after K^85^ (K^85^-3i, K^85^-7i, K^85^-9i) in the JLR. **(B,C)** Lipid mixing **(B)** and content mixing **(C)** between synaptobrevin-2 WT, K^85^-3i, K^85^-7i, and K^85^-9i liposomes and syntaxin-1/SNAP-25 liposome. **(D,E)** Lipid mixing **(D)** and content mixing **(E)** between synaptobrevin-2 WT, K^85^-3i, K^85^-7i, and K^85^-9i liposomes and Munc18-1/syntaxin-1 liposome. **(F)** Domain structure of full-length synaptobrevin-2 with 3, 7, 9 GSG insertions after L^93^ (L^93^-3i, L^93^-7i, L^93^-9i) in the JLR. **(G,H)** Lipid mixing **(G)** and content mixing **(H)** between synaptobrevin-2 WT, L^93^-3i, L^93^-7i, and L^93^-9i liposomes and syntaxin-1/SNAP-25 liposome. **(I,J)** Lipid mixing **(I)** and content mixing **(J)** between synaptobrevin-2 WT, L^93^-3i, L^93^-7i, and L^93^-9i liposomes and Munc18-1/syntaxin-1 liposome. Representative traces came from one of three independent experiments. Bars on the right panel in **(B–E,G–J)** are Means ± SDs, *n* = 3.

We increased the length of the synaptobrevin-2 JLR via inserting 3, 7, and 9 amino acids following the residue K^85^, referred to as K^85^-3i, K^85^-7i, and K^85^-9i, respectively (Figure 4A). These insertions are expected to extend the length and flexibility of the JLR outside the membrane surface, as the insertion position is upstream of W^89^/W^90^. Co-flotation assay confirmed that the insertions were reconstituted on liposomes (Supplementary Figure 1D). First, we explored the fusion effects of these insertions using the fusion system without Munc18-1 and Munc13-1, as shown in Figures 1B,C. K^85^-3i, K^85^-7i, and K^85^-9i gradually reduced lipid mixing and content mixing in a length-dependent manner (Figures 4B,C). However, in the fusion system dependent of Munc18-1 and Munc13-1, as shown in Figures 1F,G, K^85^-3i, K^85^-7i, and K^85^-9i severely impaired lipid mixing and content mixing (Figures 4D,E), consistent with the data observed in neurons (Zhou et al., 2013). Hence, the tight coupling between the SNARE motif and the TMR that is specific for evoked fusion was reproduced in *in vitro* fusion system mediated by Munc18-1 and Munc13-1, reflecting that Munc18–Munc13-dependent membrane fusion represents the dominant route leading to evoked exocytosis.

The synaptobrevin-2 JLR sequence (^86^RKYWW^90^) was recently found to bind the Munc13-1 MUN domain, and this interaction is required for the MUN domain to drive N-terminal SNARE complex assembly when starting from the Munc18-1/syntaxin-1 complex (Wang et al., 2019). Given that K^85^-3i, K^85^-7i, and K^85^-9i all retain the RKYWW sequence as an entirety (Figure 4A), we assume that the three insertions do not influence MUN activity in promoting N-terminal SNARE assembly. To test this idea, we assessed MUN activity by using established native-gel assay (Yang et al., 2015; Wang et al., 2019). As expected, synaptobrevin-2 (residues 29–93) with K^85^-3i, K^85^-7i, and K^85^-9i insertions all supported the transition from the Munc18-1/syntaxin-1 complex to the SNARE complex in the presence of the MUN domain and SNAP-25 (Supplementary Figure 5). Hence, these insertions seem unlikely to impair MUN–synaptobrevin-2 (JLR) interaction and MUN activity in N-terminal SNARE assembly. It is conceivable that the strongly impaired membrane fusion caused by K^85^-3i, K^85^-7i, and K^85^-9i insertions arises likely from the uncooperative actions among the extended synaptobrevin-2 JLR, Munc13-1/Munc18-1, and phospholipids in C-terminal SNARE assembly and membrane merge.

In addition, similar insertions were placed downstream of W^89^/W^90^, i.e., following the residue L^93^, referred to as L^93^-3i, L^93^-7i, and L^93^-9i, respectively (Figure 4F). Co-flotation assay confirmed that the insertions were reconstituted on liposomes (Supplementary Figure 1D). These insertions are assumed to increase the length and flexibility of the TMR inside the membrane. Intriguingly, these insertions had little effect on lipid mixing (Figures 4G,I), but exerted more remarkable influence on content mixing (Figures 4H,J), regardless of using the fusion systems with or without Munc18-1 and Munc13-1. These data suggest that the precise sequence length upstream of W^89^/W^90^, but not that downstream of W^89^/W^90^, accounts for the tight coupling between the SNARE motif and the TMR.

Taken together, these data suggest that Munc18-1 and Munc13-1 cooperate to impose a tight coupling demand between the SNARE motif and the TMR on membrane fusion upon specifically sensing the length of the synaptobrevin-2 JLR, leading to a notion that the JLR of synaptobrevin-2 constitutes an essential structure element determining not only force transmission but also precise regulation demanded by fast exocytosis.

## Discussion

Aromatic and charged residues are highly conserved in the juxtamembrane linker regions (JLR) of synaptobrevins and play a role in membrane fusion (Maximov et al., 2009; Williams et al., 2009; Borisovska et al., 2012; Demill et al., 2014). Two consecutive tryptophan residues (W89/W90) in the JLR of synaptobrevin-2 were reported to reside at the membrane-water interface and serve as a fusion barrier to regulate Ca2+-evoked exocytosis and membrane fusion (Kweon et al., 2003; Chen et al., 2004; Bowen and Brunger, 2006; Maximov et al., 2009; Borisovska et al., 2012). Here, we observed that helix-breaking and/or length-increasing insertions in synaptobrevin-2 exerts distinctive effects on membrane fusion, in a manner dependent on the insertion position relative to W^89^/W^90^. For the insertions downstream of W^89^/W^90^, e.g., W^90^-PP, L^93^-PP, L^93^-3i, L^93^-7i, and L^93^-9i, they exhibited ignoring effects on lipid mixing; while, for those insertions upstream of W^89^/W^90^, e.g., K^85^-PP, Y^88^-PP, K^85^-3i, K^85^-7i, and K^85^-9i, they exerted severe defects on lipid mixing. The striking differences on the influence of lipid mixing by the insertions indicates that perturbation of the sequence downstream of W^89^/W^90^ seems to be better tolerated than that upstream of W^89^/W^90^. Upon analysis and comparison of studies published previously (Mcnew et al., 1999; Van Komen et al., 2005; Kesavan et al., 2007; Pieren et al., 2015), we notice that the insertions placed after W^89^/W^90^ in the JLR indeed exhibit virtually little effect on SNARE-mediated lipid mixing, consistent well with our results. In line with the conception that W^89^/W^90^ positions at the membrane-water interface, our results suggest that W^89^/W^90^ functions as a landmark gating force transmission into the membrane interface. In our point of view, with membrane approaching by *trans*-formation of the SNARE motifs, further assembly of the JLR to W^89^/W^90^ is strictly required to initiate membrane merger. Note that helical extension of the JLR to W^89^/W^90^ is coupled with conformational change of the JLR.

Our study also examined the roles of the TMR of synaptobrevin-2 in membrane fusion. First, the observations that the insertions downstream of W^89^/W^90^, e.g., W^90^-PP, L^93^-PP, L^93^-3i, L^93^-7i, and L^93^-9i, support lipid mixing but remarkably reduce (but not eliminate) content mixing suggest that helical continuity and rigid of the TMR spanning the lipid bilayer is crucial for inner membrane merger. Similarly, replacement of the synaptobrevin-2 TMR with that of syntaxin-1 displayed an asymmetric effect on lipid and content mixing. The strong defect on content mixing caused by the replacement of the TMR might arise because specific interactions between the two heterogeneous TMRs are crucial for effective inner membrane merger, in line with previous results that the TMRs of synaptobrevin-2 and syntaxin-1 form a heterodimer during fusion (Margittai et al., 1999; Laage et al., 2000; Stein et al., 2009). Strikingly, although membrane-attached synaptobrevin-2 lacking the TMR allows *trans*-SNARE complex formation docking liposomes (Supplementary Figure 3), it totally abrogates membrane fusion regardless of the existence of Munc18-1 and Munc13s (Figures 2G,H), owing to improper location of W^89^/W^90^ with respect to the membrane caused by the deletion of the TMR (Figures 3D,E). Consistently, a recent NMR study also found that the W^89^/W^90^ residues in soluble synaptobrevin-2 (residue 1–96) are sequestered from the membrane (Lakomek et al., 2019). These data suggest that membrane proximity is not sufficient and that the TMR of synaptobrevin-2 drives membrane fusion via pulling W^89^/W^90^ at the membrane interface so as to effectively transmit the force into the membrane. In addition to serving as a force-transmission element, the SNARE TMR has been implicated in fusion pore formation (Han et al., 2004; Ngatchou et al., 2010; Chang et al., 2015; Bao et al., 2016; Sharma and Lindau, 2018; Weiss, 2019).

Notably, the observations that abrogation of fusion by helix-breaking insertions and/or by TMR deletion cannot be rescued by Munc18-1 and Munc13-1 suggest that the synaptobrevin-2 TMR is absolutely required for inducing full fusion and that Munc18-1 and Munc13-1 are unlikely to serve as independent force generators in membrane deformation and fusion. Our results are not in line with previous data that forcing lipid membranes close together suffices to induce synaptic vesicle fusion without a need for the TMR and that SM proteins Munc18-2 and SM protein-containing HOPS complex exert force to drive complete fusion regardless of the presence of the SNARE TMR (Xu et al., 2011; Zhou et al., 2013; Spessott et al., 2017). This discrepancy needs to be interpreted with caution because different lipid (protein) composition, membrane mobility and curvature, and protein concentrations, etc., were used in these studies. For instance, (i) in the presynaptic active zones, protein, and lipid components around the fusion pore are expected to be more complicate than that in *in-vitro* man-made proteoliposomes; and (ii) increasing local numbers of the SNARE complex might lead to a higher energetically favorable state that could be sufficient to destabilize the phospholipid bilayers even without other fusion components. Nevertheless, our results are consistent with a recent study showing that lipid-anchored synaptobrevin-2 provides little or no support for liposome fusion and exocytosis (Chang et al., 2016), which rationalize the virtually universal presence of a TMD in R-SNAREs (Weimbs et al., 1998; Jahn and Südhof, 1999).

Previous work has identified a tight coupling between the SNARE motif and the TMR that is specific for evoked exocytosis (Deak et al., 2006; Kesavan et al., 2007; Bretou et al., 2008; Guzman et al., 2010; Borisovska et al., 2012; Zhou et al., 2013). Our finding that Munc18-1 and Munc13-1 renders the fusion more sensitive to the length and flexibility of the synaptobrevin-2 JLR sequence upstream of W^89^/W^90^ leads to a notion that the tight coupling demanded by evoked fusion might be imposed by Munc18-1 and Munc13-1, reflecting that Munc18–Munc13-regulated SNARE complex assembly serves likely as the dominant route to evoked fusion. Actually, both spontaneous and evoke fusion require Munc18-1 and Munc13-1. Although it is believed that spontaneous and evoke fusion share same fusion machinery, the underlying mechanism may differ. In contrast to evoked fusion that relies on Munc13-catalyzed transition from the Munc18-1/syntaxin-1 complex to the SNARE complex in the presence of SNAP-25 and synaptobrevin-2, spontaneous fusion might use a different route that involves Munc18-1-mediated syntaxin-1 N-peptide interaction and Munc13-1-mediated association of the two apposing membranes. We suggest that Munc18-1 and Munc13-1 highly regulate the whole process of SNARE complex assembly in evoked fusion, whereas their predominant roles in spontaneous fusion lie in vesicle docking and membrane association prior to SNARE assembly. Increasing evidence has indicated that the JLR of synaptobrevin-2 serves as an important element not only for force transmission but also for regulations by multiple fusion components such as calmodulin, Munc13s, and phospholipids (Quetglas et al., 2000; Kweon et al., 2003; Bowen and Brunger, 2006; Ellena et al., 2009; Brewer et al., 2011; Borisovska et al., 2012; Han et al., 2016; Rathore et al., 2019; Wang et al., 2019). Although length-increasing insertions in the synaptobrevin-2 JLR do not disturb Munc13-mediated N-terminal SNARE assembly (Supplementary Figure 5), structural perturbations in this region may affect cooperative interplay among the JLR, Munc13s, and phospholipids, therefore altering fusion competence. For instance, the crystal structure of synaptobrevin-2 bound to Munc13-1 reveals a rigid but non-typical α-helical conformation of the JLR (Wang et al., 2019), suggesting that kinetics and thermodynamics for the conformational change of the JLR from priming to fusion may differ between the two fusion system with and without Munc13-1. Interestingly, a recent study showed that vacuoles in yeast are connected by a metastable, non-expanding, nanoscopic fusion pore that does not allow passage of some cargo. This work suggests that this is the default state, from which full fusion is regulated and that the SNARE TMR and the SM protein-containing HOPS complex stabilize the pore against re-closure (D'Agostino et al., 2018). Hence, it is also likely that the tight coupling imposed by Munc18-1 and Munc13-1 in evoked fusion might be due to the existence of small and dynamic fusion pores whose expansion can be regulated and stabilized by interactions of the synaptobrevin-2 JLR with Munc13-1 and/or Munc18-1. These may account for differentiated demand of the tight coupling between spontaneous and evoked fusions. Finally, the effects of Munc18-1 and Munc13-1 found in present study might depend partly on interplay with the synaptotagmin-1 C_2_AB and Ca^2+^, because of their existence in all our reactions. Despite these speculations, future investigations need to test how the regulatory fusion components selectively manipulate the structure and function of the JLR in different types of exocytosis.

## Materials and Methods

### Plasmids Construction

All full-length synaptobrevin-2 mutant constructs were generated by PCR using the overlap expansion method. For synaptobrevin-2 R^86^-PP, Y^88^-PP, W^90^-PP, L^93^-PP variants, forward and reverse overlap primer encoding 2 proline (CCTCCG) were inserted into the target site. The same procedure was used to create R^85^-3i and L^93^-3i (GSG), R85-7i and L^93^-7i (GSGTGSG), R^85^-9i and L^93^-9i (GSGGSGGSG) insertion constructions and Syb^Syx−TMR^. All above PCR fragments were cut by BamHI (5′) and EcoRI (3′) (all from Thermo Fisher Scientific; America) restriction enzyme and then ligated into pGEX-6P-1 vector (GE Healthcare; Piscataway, NJ). To create Syb^ΔTMR^, PCR amplification of synaptobrevin-2 (residues 23–93) with a stop primer encoding six Histidine at the C-terminal was subcloned into pET-28a (Novagen; Australia) by NcoI and XhoI.

### Protein Purification

Full-length synaptobrevin-2 and its recombinant variants were expressed as N-terminal GST fusion proteins (pGEX-6P-1-vector) in the *E.coli* strain BL21-DE3 and purified using glutathione-agarose in 1% (w/v) n-octyl β-D-glucopyranoside (Amresco; Solon, OH). All column elutions were analyzed for integrity and purity of the expressed proteins by SDS-PAGE and Coomassie blue staining. Syntaxin-1 (residues 183–288, the SNARE motif, JLR and TMR), SNAP-25 (with its four cysteines mutated to serines), Munc18-1/syntaxin-1(residues 1–288, full-length), Munc13-1 (the C_1_-C_2_B-MUN fragment, residues 529–1407, EF, 1453–1531), and the synaptotagmin-1 cytoplasmic domain (C_2_AB, residues 140–421) were expressed and purified as described previously (Sutton et al., 1998; Dulubova et al., 1999, 2007; Ma et al., 2011, 2013). Protein concentrations were determined by UV-visible spectrometer (SHIMADZU UV-2450).

### Lipid Mixing Assay

Proteoliposomes were prepared using established procedures (Ma et al., 2013). Donor (synaptobrevin-2) liposomes contained 1-Palmitoyl-2-oleoyl-sn-glycero-3-phosphocholine (POPC), 1-palmitoyl-2-oleoyl-sn-glycero-3-phosphoethanolamine (POPE), 1,2-dioleoyl-sn-glycero-3-phospho-L-serine (sodium salt) (DOPS), 1,2-dipalmitoyl-snglycero-3-phosphoethanolamine-N-[lissamine rhodamine B sulfonyl] ammonium salt (rhodamine-PE), 1,2-dipalmitoyl-sn-glycero-3-phosphoethanolamine-N-[7-nitro-2-1,3-benzoxadiazol-4-yl] ammonium salt (NBD-PE). Acceptor liposome (syntaxin-1/SNAP-25) contained 60% POPC, 20% POPE, 20% DOPS, and the other acceptor liposome (Munc18-1/syntaxin-1) contained additional 2% L-α-phosphatidylinositol-4,5-bisphosphate (Brain, Porcine) (ammonium salt) (PI[4, 5]P2) and 5% 1-2-dioleoyl-sn-glycerol (DAG) (all from Avanti Polar Lipids; Alabaster, AL). Lipid mixtures were dried in glass tubes with nitrogen gas and followed by vacuum for at least 3 h. Lipid films were resuspended in buffer A [25 mM HEPES, pH 7.4, 150 mM KCl, 10 % glycerol (v/v)] and 1 mM DTT, 1% 3-[(3-Cholamidopropyl) dimethylammonio] propanesulfonate (CHAPS) (w/v, Amersco) and vortexed for 10 min. Purified proteins were added slowly to the micelle at a final concentration of 5 mM total lipids. Liposome was acquired with a constant protein/lipid ratio which acceptor liposome is 1:800 and donor liposome is 1:500. The liposome protein mixtures were incubated at room temperature for 30 min followed by dialyzing in buffer A and 1 mM DTT, 1 g/L Bio-beads SM2 (Bio-Rad) 3 times at 4°C in order to remove the detergent extensively. Lipid mixing assay was then taken up based on 7-nitrobenz-2-oxa-1,3-diazole (NBD) fluorescence dequenching assay which is emitted at 538 nm and excited at 460 nm. In brief, Donor liposomes (0.25 mM lipids) were mixed with acceptor liposomes (0.5 mM lipids) in the presence of 2 μM C_2_AB fragment, 0.5 mM Ca^2+^ (syntaxin-1/SNAP-25) and extra 1 μM Munc13-1 C_1_-C_2_B-MUN, 5 μM SNAP-25 (Munc18-1/syntaxin-1) (as indicated in the figures) in a total volume of 60 ul. All experiments were performed at 30°C. At the end of each reaction, 1% w/v β-OG was added to solubilize the liposomes and obtain maximum fluorescence signal for normalizing.

### Content Mixing Assay

Forty millimeters sulforhodamine B (Sigma) was loaded into synaptobrevin-2 liposome without lipid probes. Other experimental details were same as lipid mixing assays. leakiness control was performed with 40 mm sulforhodamine B both loaded into syntaxin-1/SNAP-25 or Munc18-1/syntaxin-1 liposome and synaptobrevin-2 liposomes. fluorescence was monitored on a PTI QM-40 fluorescence spectrophotometer with an excitation wavelength of 565 nm and an emission wavelength of 580 nm. Fluorescence normalization is the same as that used in the lipid-mixing assay. All experiments were carried out at 30°C. At the end of each reaction, 1% w/v β-OG was added to solubilize the liposomes and obtain maximum fluorescence signal for normalizing.

### Liposome Co-flotation Assay

Lipid films were re-suspended in buffer A [25 mM HEPES, pH 7.4, 150 mM KCl, 1 mM DTT, 10% glycerol (v/v)] and vortexed for at least 5 min. The re-suspended lipid films were frozen and thawed five times, and then extruded through a 50 nm polycarbonate filter with an Avanti extruder for at least 29 times to make the final liposome. Syb^ΔTMR^ was purified as the C-terminal 6-Histidine tagged tail-anchored forms earing a DGS-NTA lipid 1,2-dioleoyl-sn-glycero-3-[(N-(5-amino-1-carboxypentyl) iminodiacetic acid) succinyl] (nickel salt) that can be attached to lipid membranes. Proteins were preincubated with liposomes made by Extruder with a protein to lipid ratio of 1:100 for 3 h at room temperature. Co-floatation assay was firstly taken with a Histodenz (Sigma Aldrich) gradient density gradients (40:30%) using a SW 55 Ti rotor (Beckman Coulter; Boulevard Brea, CA) at a speed of 48,000 rpm for 2 h in order to get rid of remaining unattached 6-Histidine tail-anchored proteins. Samples from the top and the bottom of the gradient (50 μl) were taken and analyzed by SDS-PAGE and Coomassie Blue staining.

### Fluorescence Anisotropy Assay

Synaptobrevin-2 (residues 49–96, S61C) was purified and labeled with BODIPY FL N-(2-aminoethyl)-maleimide (BDPY) according to the manufacturer's instruction (Molecular Probes). Syntaxin-1 (residues 183–288), SNAP-25 and synaptobrevin-2 (residues 49–96, S61C) were first preincubated at a molar ratio of 1:1:1 at room temperature for 3 h. Liposome were acquired using dialysis method described above. Co-floatation assay was then conducted to get rid of excess fluorescence labeled synaptobrevin-2. Syntaxin-1/SNAP-25/synaptobrevin-2 49–96 S61C liposome (0.25 mM lipids) was mixed with Syb^ΔTMR^ liposome (0.5 mM lipids) and the fluorescence anisotropy assay was performed on PTI QM-40 fluorescence spectrophotometer equipped with a set of polarizers. The experiment was carried out at 25°C with excitation and emission wavelength of 485 and 513 nm, respectively.

### Tryptophan Fluorescence Quenching Assays

To explore the location of tryptophan residues in synaptobrevin-2 WT and mutants, lipid quencher 1-palmitoyl-2-stearoyl-(4.5)-dibromo-sn-glycero-3-phosphocholine (4,5-Br_2_-PC, 10 mg/ml) was added in replacement of part of POPC at 0.0, 0.1, 0.2, 0.3, 0.4 molar fraction, respectively. Solutions of POPC (14 mg/ml) and 4,5-Br_2_-PC (10 mg/ml) in chloroform were codissolved at 5 mM total lipid. Proteins were reconstituted into the liposome with a molar lipid/protein ratio of 250 using dialysis method described above. The degree of quenching was determined as a function of the mole fraction of added brominated POPC. The samples were excited at 285 nm, and the emission spectra were collected in the range of 300–400 nm. The total fluorescence intensity F was obtained by integrating the intensity in this spectral range. Normalized statistics method was described elsewhere (Kweon et al., 2003).

### Native Gel Assay

Munc18-1 and syntaxin-1 (residues 2–253) were first incubated with a protein/protein ratio of a 1.2:1 at 30°C for 3 h to form a stable Munc18-1/syntaxin-1 (M18/Syx) complex. Ten micrometre synaptobrevin-2 (residues 23–93) or its mutants, 10 μM SNAP-25, 25 μM MUN (residues 933–1407, EF, 1453–1531) were then added and incubated for another 3 h at 30°C. The samples were loaded into the non-denaturing [sodium dodecyl sulfate (SDS)-free] gel and the electrophoresis were performed in native electrophoresis buffer at 4°C as described before (Yang et al., 2015). The representative gel displayed is from one of three replicates.

### Data Analysis

Prism 6.01 (GraphPad) was used for graphing and performing linear regression.

## Data Availability Statement

The original contributions presented in the study are included in the article/Supplementary Materials, further inquiries can be directed to the corresponding author/s.

## Author Contributions

YH and LZ generated all mutants and performed and analyzed *in vitro* fusion experiments. YH and CM wrote the manuscript. CM conceived the experiments and supervised the study. All authors contributed to the article and approved the submitted version.

## Conflict of Interest

The authors declare that the research was conducted in the absence of any commercial or financial relationships that could be construed as a potential conflict of interest.

## Abbreviations

JLR: Juxtamembrane Linker Region
TMR: transmembrane region
Syb2: Synaptobrevin-2
M18/Syx: Munc18-1/syntaxin-1
POPC: 1-Palmitoyl-2-oleoyl-sn-glycero-3-phosphocholine
POPE: 1-palmitoyl-2-oleoyl-sn-glycero-3-phosphoethanolamine
DOPS: 1,2-dioleoyl-sn-glycero-3-phospho-L-serine (sodium salt)
rhodamine-PE: 1,2-dipalmitoyl-snglycero-3-phosphoethanolamine-N-[lissamine rhodamine B sulfonyl] ammonium salt
NBD-PE: 1,2-dipalmitoyl-sn-glycero-3-phosphoethanolamine-N-[7-nitro-2-1,3-benzoxadiazol-4-yl] ammonium salt
PI[4,5]P2: L-α-phosphatidylinositol-4,5-bisphosphate (Brain, Porcine) (ammonium salt)
DAG: 1-2-dioleoyl-sn-glycerol
DGS-NTA: 1,2-dioleoyl-sn-glycero-3-[(N-(5-amino-1-carboxypentyl) iminodiacetic acid) succinyl] (nickel salt)
BDPY: BODIPY FL N-(2-aminoethyl)-maleimide.
