# Supplementary material for: Structural Roles for the Juxtamembrane Linker Region and Transmembrane Region of Synaptobrevin 2 in Membrane Fusion
Source: Front Cell Dev Biol. 2021 Jan 6;8:609708. doi: 10.3389/fcell.2020.609708 (PMC7815645; doi:10.3389/fcell.2020.609708)
Supplement: Supplementary file 1 [file Table_1.DOCX]

Supplementary Material

**Supplementary Figures**

**
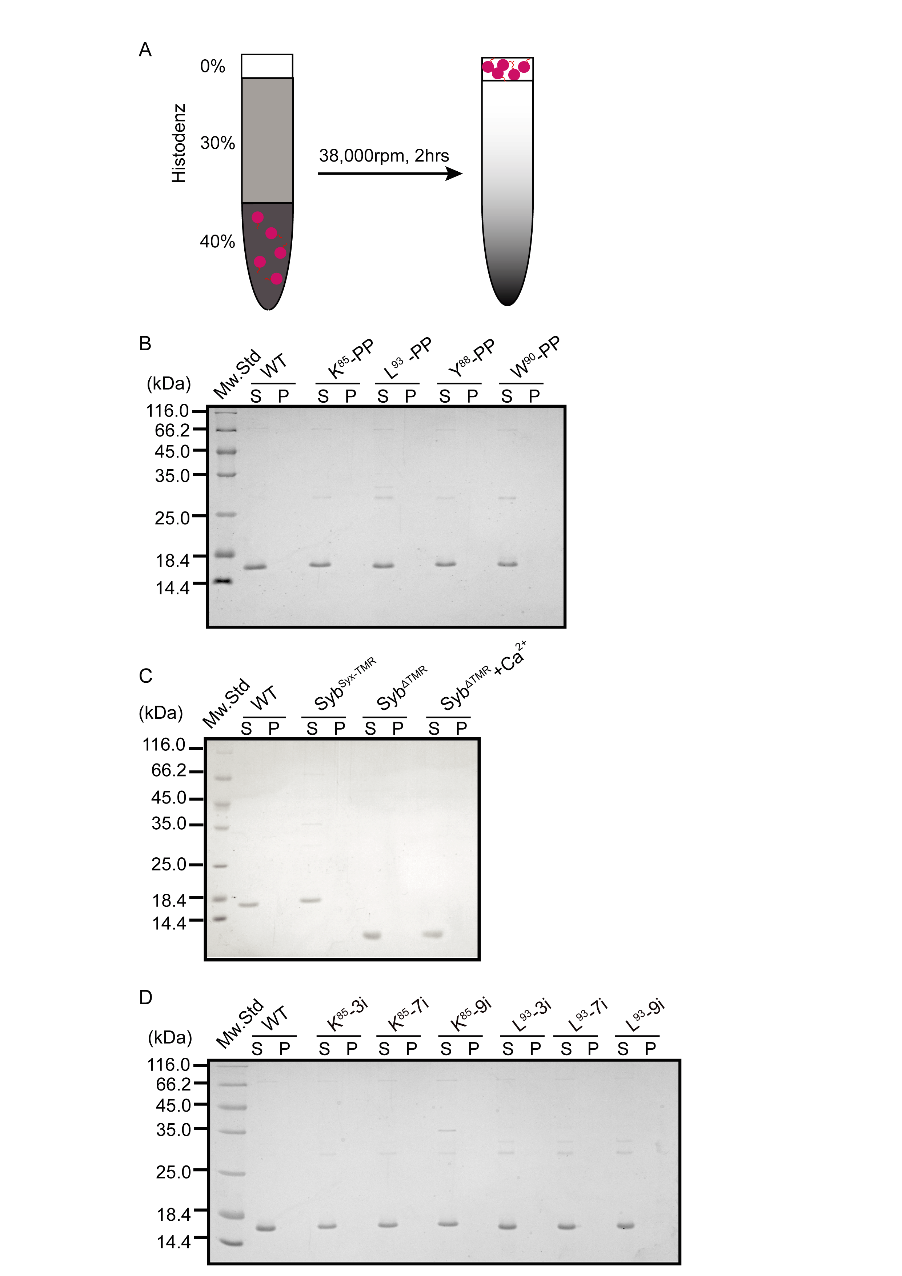
**

**Supplementary Figure 1.** **All synaptobrevin-2 mutants were able to bind to the membrane.** (**A**) Schematic diagram of the liposome co-flotation assay. After centrifuging, liposomes and bound proteins (fuchsia) were co-floated on the top of the density gradients, remaining unbounded proteins left in the bottom of the gradients. (**B**) Analysis of co-flotation assay of synaptobrevin-2 WT, K^85^-PP, L^93^-PP, Y^88^-PP and W^90^-PP by SDS-polyacrylamide gel electrophoresis and Coomassie Brilliant Blue staining. S, supernatant; P, pellet. (**C**) Analysis of co-flotation assay of synaptobrevin-2 WT, Syb^Syx-TMR^ and Syb^∆TMR^ and Syb^∆TMR^ with 1mM CaCl_2_ by SDS-polyacrylamide gel electrophoresis and Coomassie Brilliant Blue staining. (**D**) Analysis of co-flotation assay of synaptobrevin-2 WT, K^85^-3i, K^85^-7i, K^85^-9i, L^93^-3i, L^93^-7i and L^93^-9i by SDS-polyacrylamide gel electrophoresis and Coomassie Brilliant Blue staining.

**
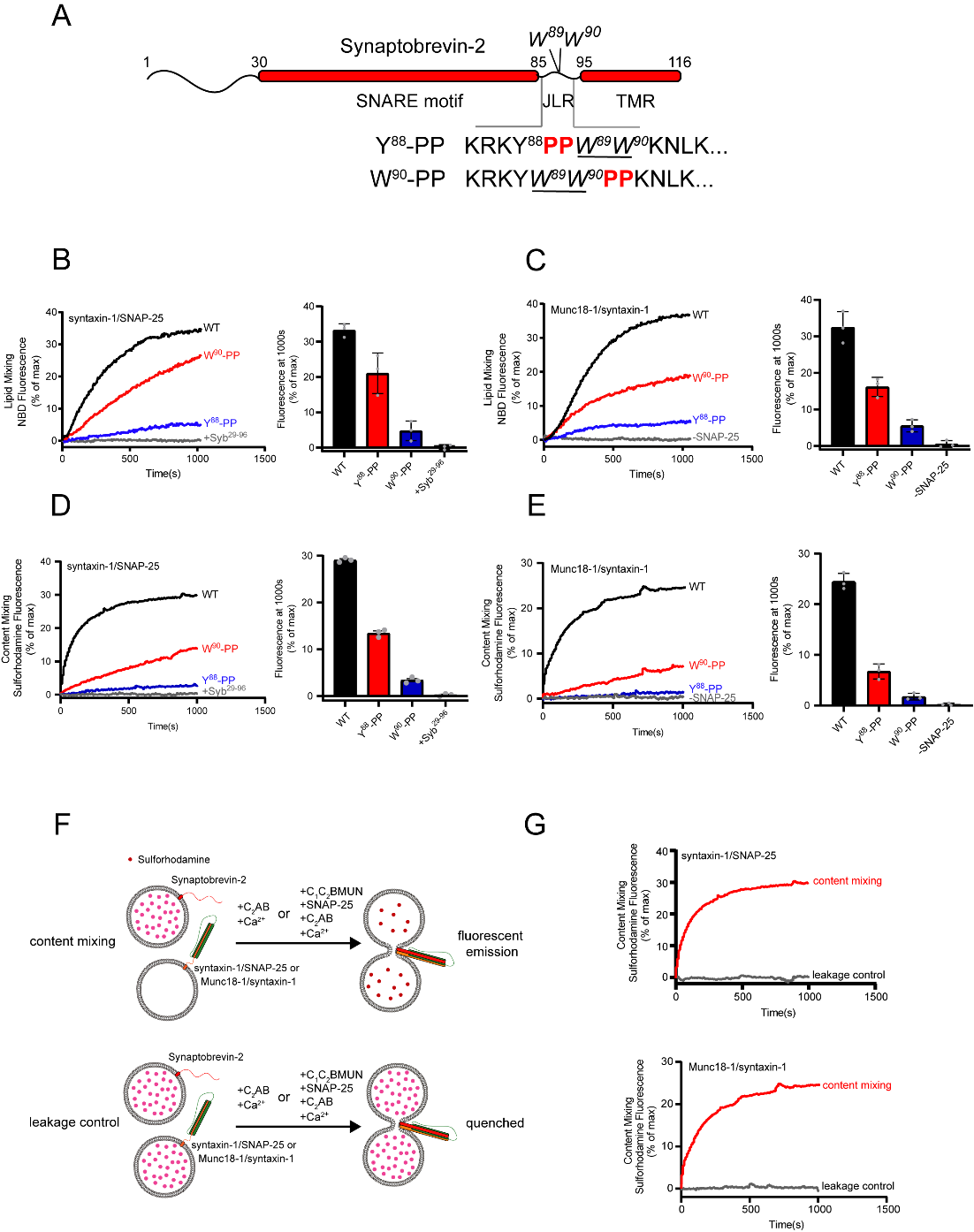
**

**Supplementary Figure 2. Fusion affected by disrupting helical continuity of the synaptobrevin-2 adjacent to W^89^/W^90^**. (**A**) Domain structure of full-length synaptobrevin-2 with two proline insertions after Y^88^ (Y^88^-PP) or W^90^ (W^90^-PP) in the JLR. (**B, C**) Lipid (**B**) and content mixing (**C**) of synaptobrevin-2 WT, Y^88^-PP and W^90^-PP liposomes with syntaxin-1/SNAP-25 liposomes. (**D, E**) Lipid (**D**) and content mixing (**E**) between Munc18-1/syntaxin-1 (full length, residues 1–288) and synaptobrevin-2 liposomes in the presence of the Munc13-1 C1-C2B-MUN fragment, SNAP-25, C_2_AB fragment and 1 mM Ca^2+^. Representative traces came from one of three independent experiments. Bars on the right panel in (**B**), (**C**), (**D**) and (**E**) are Means ± SDs, n = 3. (**F**) Schematic diagram of the normal content mixing and the leakage control assays. In the leakage control, both liposomes were loaded with 40 mM sulforhodamine. (**G**) Leakage was not detected in both content mixing of synaptobrevin-2 WT with syntaxin-1/SNAP-25 liposomes or with Munc18-1/syntaxin-1 liposomes.

**
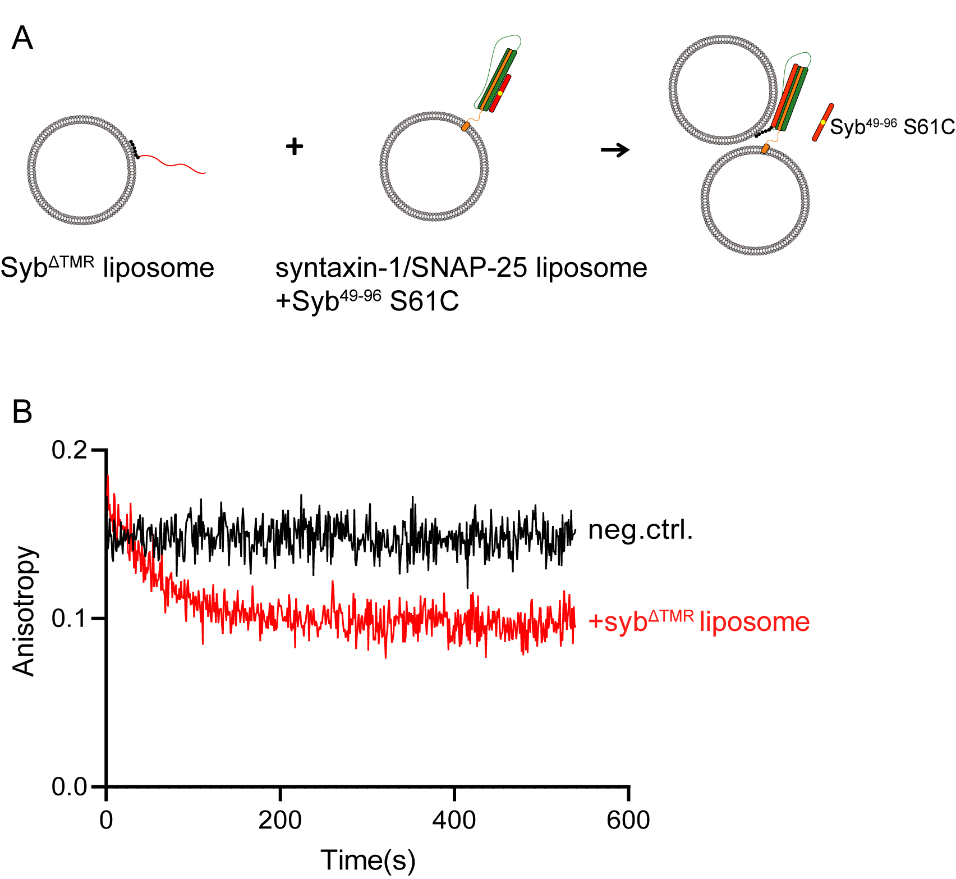
**

**Supplementary Figure 3. Syb^∆TMR^ failed to support liposome fusion but retained the ability to form the SNARE complex.** (**A**) Schematic depiction of the SNARE complex formation between Syb^∆TMR^ liposome and syntaxin-1/SNAP-25 liposome monitored by fluorescence anisotropy. Syntaxin-1 (residues 183–288) and SNAP-25 were mixed with BDPY-labeled synaptobrevin-2 (residues 49–96, S61C, Syb^49-96^ S61C) at a molar ratio of 1:1:1 and incubated at room temperature for 3 hours. Co-flotation assay was then conducted to get rid of excess fluorescence labeled synaptobrevin-2. (**B**) Detection of the SNARE complex formation between syntaxin-1/SNAP-25 liposome and Syb^∆TMR^ liposome by ﬂuorescence anisotropy. Upon complete zippering of Syb^∆TMR^ with syntaxin-1/SNAP-25, release of Syb^49-96^ S61C from syntaxin-1/SNAP-25 liposome led to a decrease in anisotropy.

**
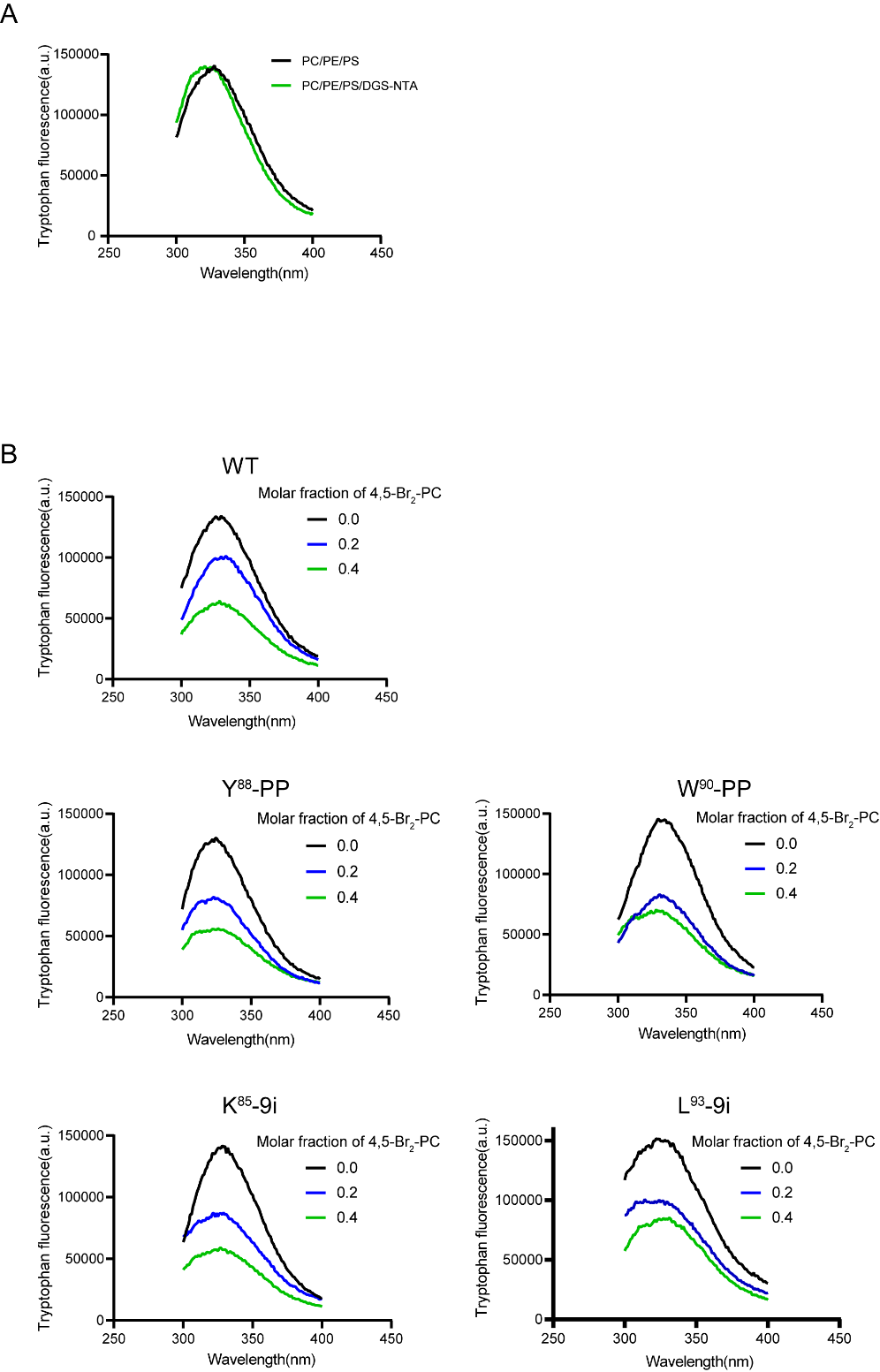
**

**Supplementary Figure 4. The behavior of tryptophan quenching using different types of liposomes and synaptobrevin-2 mutants.** (**A**) 5% molar ratio of DGS-NTA did not quench tryptophan fluorescence of synaptobrevin-2 WT. Synaptobrevin-2 WT was reconstituted in different liposomes in the presence or absence of 5% DGS-NTA. No quenching of tryptophan was observed in PC/PE/PS/DGS-NTA liposome compared with PC/PE/PS liposome. (**B**) The longest Glycine/Serine insertions (K^85^-9i and L^93^-9i) and the nearest proline insertions adjacent to W^89^/W^90^ (Y^88^-PP and W^90^-PP) still displayed a certain extent of decreased tendency of tryptophan fluorescence when 4,5-Br_2_-PC concentration increased (Molar fraction of 4,5-Br_2_-PC: 0.0, 0.2, 0.4). Samples in (**A**) and (**B**) were all excited at 285 nm, and the emission spectra were collected in the range of 300-400 nm.


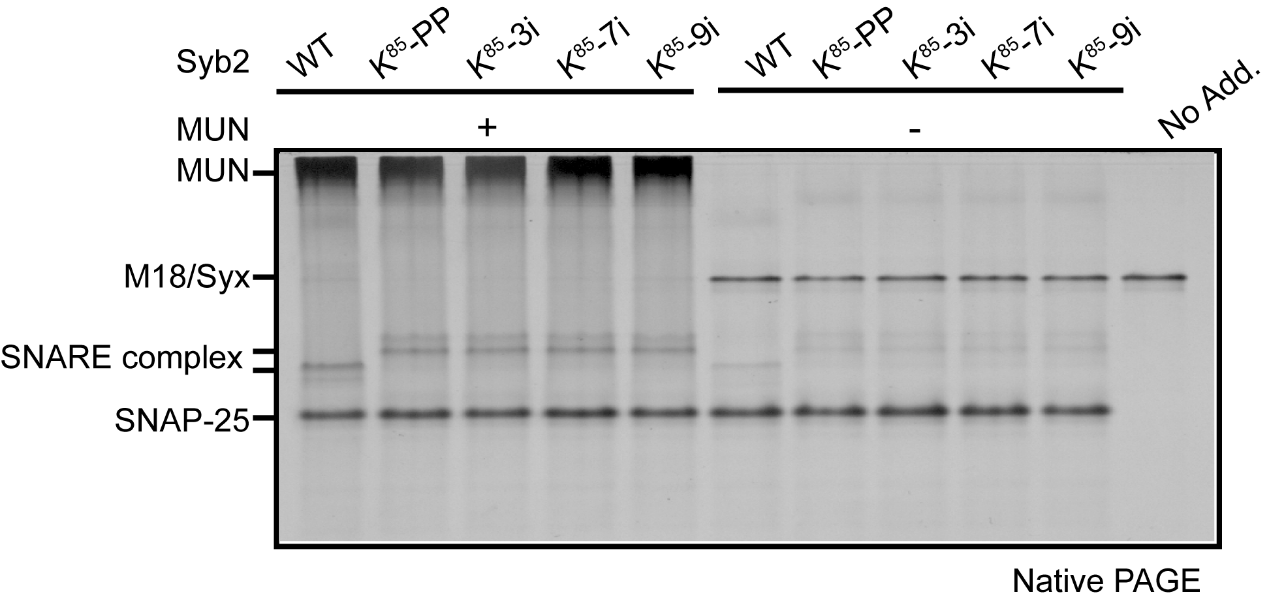


**Supplementary Figure 5. K^85^ mutants did not affect the transition of Munc18-1/syntaxin-1 complex to SNARE complex.** Native PAGE assay for monitoring MUN (residues 933–1407, EF, 1453–1531)-catalyzed transition from the Munc18-1/syntaxin-1 (residues 2–253) complex (referred to as M18/Syx) to the SNARE complex. The M18/Syx (2 μM) displayed a sharp band at the top of the gel; upon the addition of the MUN (25 μM), SNAP-25 (10 μM), and synaptobrevin-2 (residues 23–93, Syb2) WT or its mutants (K^85^-PP, K^85^-3i, K^85^-7i and K^85^-9i) (10 μM), this band disappeared with the formation of the SNARE complex. Disappearance of the M18/Syx complex can only be detected when all components were included. The representative gel displayed came from one of three replicates.
